# Efficacy and Safety of Tirofiban for the Management of Acute Ischemic Stroke: A Systematic Review and Meta‐Analysis of Randomized Controlled Trials (RCTs)

**DOI:** 10.1002/brb3.71520

**Published:** 2026-05-31

**Authors:** Dorothy Lim Bullecer, Cara Mohammed, Julie Anne De Lima Loiola, Mary Gabrielle Te Chy, Samratul Fuadah Ramli, Abimbola Sodiq Hussein, Wasid Bin Showkat, Thahsin Taikadan, Sarika Mutyala, Muhammad Ayyan, Muhammad Aemaz Ur Rehman, Adeel Ahmad, Asma'a Munasar Ali Alsubari

**Affiliations:** ^1^ Department of Medicine Garcia Memorial Provincial Hospital Bohol Philippines; ^2^ Department of Orthopaedic Surgery Sangre Grande Hospital Sangre Grande Trinidad and Tobago; ^3^ Department of Medicine Mato Grosso do Sul Regional Hospital Mato Grosso do Sul Brazil; ^4^ Department of Medicine Allied Care Experts Medical Center Cebu Philippines; ^5^ Department of Medicine Batterjee Medical College Jeddah Saudi Arabia; ^6^ Department of Medicine Raigmore Hospital Inverness UK; ^7^ Department of Medicine Government Medical College Baramulla Jammu and Kashmir India; ^8^ Department of Medicine Government Medical College, Kozhikode Kerala India; ^9^ Department of Medicine Gandhi Medical College Hyderabad India; ^10^ Department of Medicine King Edward Medical University Neela Gumbad Lahore Pakistan; ^11^ Department of Neurology University of Alabama Tuscaloosa Alabama USA; ^12^ Department of Cardiovascular Medicine Mayo Clinic Rochester New York USA; ^13^ Faculty of Medicine Sana'a University Sana'a Yemen

**Keywords:** ischemic stroke, meta‐analysis, stroke, tirofiban

## Abstract

**Background**: Tirofiban is a GP IIb/IIIa inhibitor, an anti‐platelet drug that is used in ischemic stroke or acute coronary syndromes. The goal of this study is to assess the safety and efficacy of the drug in patients who have suffered an acute ischemic stroke.

**Methods**: A comprehensive search of PubMed, Embase, the Cochrane Library, and ClinicalTrials.gov from inception to December 2025 yielded randomized controlled trials (RCTs) comparing Tirofiban and conventional management in patients with acute ischemic stroke. We assessed the risk of bias using the revised Cochrane “Risk of bias” tool for randomized trials (RoB 2.0). We analyzed the outcomes using RevMan 5.4, with risk ratio (RR) as the effect measure.

**Results**: A total of 9 RCTs with 4210 patients were included in our meta‐analysis. According to our meta‐analysis, the tirofiban group was associated with a statistically significant increase in the patients showing functional independence at 90 days, measured by patients with Modified Rankin Score (mRS) of 0–2 (RR 1.13; CI = 1.06–1.20) and excellent outcome (mRS score 0–1) at 90 days (RR 1.17; CI = 1.05–1.29). We found no statistically significant difference between the two groups regarding mortality (RR 0.94; CI = 0.63–1.40). The tirofiban group had an increased incidence of intracranial hemorrhage (ICH) (RR 1.25; CI = 1.06–1.47) but had a similar incidence of symptomatic ICH as compared to the control group (RR 1.48; CI = 0.98–2.24).

**Conclusion**: This meta‐analysis suggests that tirofiban may improve favorable functional outcomes (mRS 0–2) in acute ischemic stroke, although the certainty of evidence was moderate. While no significant increase in sICH was observed, some analyses showed a higher rate of overall intracranial hemorrhage. These findings should therefore be interpreted cautiously, and further high‐quality randomized trials are needed to confirm the efficacy and safety of tirofiban before routine guideline use.

## Graphical abstract

Graphical representation of the comparative efficacy and safety of intravenous tirofiban for acute ischemic stroke in randomized controlled trials.

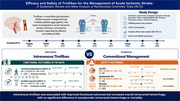

## 1 Introduction

Acute ischemic stroke (AIS) is characterized by insufficient blood supply to the brain, usually as a result of an acute thrombus or a stenotic artery (Hui et al. 2024). It leads to high rates of mortality and is the second leading cause of death worldwide (Tadi and Lui 2024). In addition, acute stroke is associated with a significant burden of disability, and thus, timely intervention is essential to increasing the chances of neurological recovery, enhancing clinical outcomes, and reducing disability in stroke patients (Sacco et al. 2013).

Current established treatments for AIS include intravascular thrombolysis (IVT) (such as the use of alteplase or tenecteplase) and endovascular therapy (EVT) such as mechanical thrombectomy (Goyal et al. 2016). Mechanical thrombectomy is an endovascular procedure that allows for rapid arterial recanalization and has been demonstrated to be superior to pharmacological therapy when performed in appropriately selected patients (Bracard et al. 2016). Additionally, the combination of these treatments has been shown to improve patient prognosis with increased functional independence as opposed to using IVT alone. Nevertheless, these treatments still pose a risk for failed reperfusion or re‐occlusion in stroke patients, mainly due to platelet aggregation and endothelial injury during the thrombectomy procedure (Goyal et al. 2016).

An emerging point of interest in stroke treatment is the safety and efficacy of tirofiban. Tirofiban is a glycoprotein IIb/IIIa receptor antagonist that prevents both platelet aggregation and thrombosis via blockage of their common pathway (Winter and Juergens 2008). It has been used extensively in the treatment of atherosclerotic heart disease due to its improved safety profile in comparison with other drugs such as abciximab and eptifibatide. Tirofiban's lower risk of bleeding can be attributed to its rapid onset of action, short half‐life, low protein‐binding rate, and receptor reversibility (Liu et al. 2023). Due to these factors, it is thought to be a potential therapeutic intervention in stroke patients and several trials have been done to investigate this (Qiu et al. 2022; Siebler et al. 2011; Wenjie et al. 2023; Torgano et al. 2010; Yu et al. 2022; Han et al. 2022; Zhang et al. 2022; Du et al. 2022). Despite these trials showing a potential benefit in terms of functional outcome and mortality, there remains uncertainty regarding other outcomes such as consequent intracerebral hemorrhage (Cai et al. 2022). Moreover, the FDA has not approved the use of tirofiban for AIS and its approval currently encompasses mainly acute coronary syndromes such as myocardial infarction and unstable angina. Therefore, the routine recommendation of tirofiban in acute ischemic stroke is yet to be established and this meta‐analysis is thus conducted to evaluate the efficacy and safety of tirofiban in AIS patients.

Previous studies comprised both observational studies such as cohorts and experimental studies such as trials, whereas this meta‐analysis aims to assess solely the results of previous randomized controlled trials. Furthermore, there have been additional trials conducted such as the ASSET‐IT trial (Tao et al. 2025) and RESCUE‐BT2 trial that is included in this meta‐analysis. Thus, this study aims to provide a more current and comprehensive systematic review of the efficacy and safety of tirofiban in cases of acute ischemic stroke.

## 2 Materials and Methods

This systematic review was conducted according to the guidelines of the Cochrane Handbook for Systematic Reviews of Interventions (Higgings et al. 2023) and reported according to the Preferred Reporting Items for Systematic Reviews and Meta‐Analysis (PRISMA) guidelines (Page et al. 2021). This review has been registered with the International Prospective Register of Systematic Reviews (PROSPERO) under the identifier CRD42024565762. Our study did not require ethical approval.

### 2.1 Eligibility Criteria

The inclusion criteria were as follows: (1) the study population included individuals with acute ischemic stroke who were eligible to undergo tirofiban therapy; (2) the intervention was tirofiban treatment; (3) the comparator was standard treatment; and (4) the outcomes included functional outcomes using the modified Rankin Score, mortality, any ICH and symptomatic ICH. We aimed to include all randomized controlled trials irrespective of publication status. Studies were excluded if they were non‐randomized in design, including quasi‐randomized or observational studies, or if they were conducted in animal models. For consistency in intervention type, only studies evaluating intravenous tirofiban as primary medical therapy for acute ischemic stroke were included. Studies in which tirofiban was administered as bridging therapy prior to endovascular thrombectomy or as rescue therapy during EVT were excluded from the analysis.

### 2.2 Information Sources

We searched the following electronic databases and international trial registers from inception to December 2025 with no language restrictions: Cochrane Central Register of Controlled Trials (via the Cochrane Library), MEDLINE (via PubMed), Embase (via Ovid), Clinical‐Trials.gov along with gray literature sources such as ProQuest Dissertations and Theses Global (PQDT), and OpenGrey to identify additional relevant data. The reference lists of included articles and relevant systematic reviews were screened to find other potentially eligible studies. We also performed citation tracking using the Web of Science to identify other potentially eligible studies. The detailed search strategy is presented in Table S1.

### 2.3 Selection Process

All the retrieved articles were imported into Rayyan and deduplication and screening of all the articles was done. Deduplication was performed by 2 reviewers independently and then the title and abstract screening was carried out by two authors independently. Any disagreements between the two authors were resolved by discussion or by a third author. Subsequently, the full‐text screening was carried out.

### 2.4 Data Collection Process and Data Items

After the process of study selection, a pre‐piloted Excel spreadsheet was used for the data extraction. Two review authors independently extracted the data from the articles and one author reviewed the data extraction. Relevant data items were extracted including patient characteristics (age, number of patients with hypertension, the incidences of hyperlipidemia, coronary artery disease, diabetes mellitus, smoking, history of anticoagulation, history of antiplatelet use, mean NIHSS score and localization of presenting deficit), intervention details (follow‐up period), comparator details, study characteristics (e.g., study design, trial name, first author, name of the country of recruited patients, number of patients), and the outcome variables.

### 2.5 Outcomes

Our primary outcome was the proportion of patients achieving functional independence using the modified Rankin scale (mRS score 0–2) at 90 days. Our secondary outcomes included the excellent outcome (mRS score 0–1) at 90 days, mortality, any intracranial hemorrhage (ICH), and symptomatic ICH (sICH).

### 2.6 Risk of Bias Assessment

Risk of bias was evaluated using the Revised Cochrane Risk of Bias tool for randomized trials (RoB 2.0) across five domains: the randomization process, deviations from intended interventions, missing outcome data, outcome measurement, and selection of reported results. Two reviewers independently assessed each study as low risk, high risk, or having some concerns, with disagreements resolved by a third reviewer.

### 2.7 Data Synthesis

Review Manager (RevMan, version 5.4; The Cochrane Collaboration, Denmark) was used for the statistical analysis. The random‐effects model was used in all analyses. Dichotomous outcomes were reported as relative risk ratios (RR) with 95% confidence intervals (CIs). We evaluated for heterogeneity using the Chi^2^ test and calculated the *I* ^2^ statistic to quantify the heterogeneity.

### 2.8 Certainty of Evidence Assessment

Two authors independently evaluated the certainty of the evidence using the five GRADE domains: study limitations, consistency of effect, imprecision, indirectness, and publication bias (Guyatt et al. 2008). Pooled estimates were considered imprecise if the optimal information size was not met or if the 95% confidence intervals included both the null effect and clinically important benefit or harm (Guyatt et al. 2011). The overall certainty of evidence was graded as high, moderate, low, or very low. High certainty indicates that further research is unlikely to change confidence in the effect estimate, while moderate certainty suggests it may have an important impact. Low certainty implies that further studies are likely to affect the estimate, and very low certainty indicates considerable uncertainty. Results are summarized in Table 3.

## 3 Results

### 3.1 Included Studies

**FIGURE 1:**
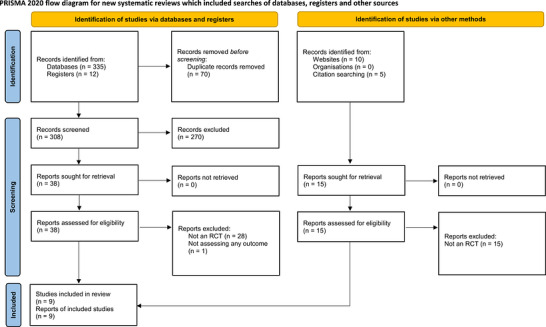
PRISMA 2020 flow chart. Flow chart of included and excluded trials. PRISMA, Preferred Reporting Items for Systematic Reviews and Meta‐Analyses.

After searching of databases, a total of 335 studies were retrieved and imported into Rayyan. Furthermore, 15 studies were obtained from gray literature sources. After deduplication, title and abstract screening, and full‐text screening, a total of nine RCTs were included in our meta‐analysis (Qiu et al. 2022; Siebler et al. 2011; Wenjie et al. 2023; Torgano et al. 2010; Yu et al. 2022; Han et al. 2022; Zhang et al. 2022; Du et al. 2022; Tao et al. 2025). The PRISMA flowchart is presented in Figure 1.

### 3.2 Study Characteristics

A total of 4210 patients were studied in the nine RCTs included in this meta‐analysis. In the intervention group, there were 2135 patients whereas there were 2075 patients in the control group. More than half of the participants in the intervention group (59.17%) and control (58.60%) were males. Apart from one study by Siebler et al. (2011) that had a follow‐up duration of 5 months, all studies had a follow‐up duration of 90 days. The characteristics of the included studies are presented in Tables 1 and 2.

**TABLE 1:** Study characteristics of the included studies.

Study authors	Sample size		Mean age		Male (%)		Follow‐up period	Hypertension (%)		Hyperlipidemia		Coronary artery disease		Diabetes mellitus		Smoking	
	Tirofiban	Control	Tirofiban	Control	Tirofiban	Control		Tirofiban	Control	Tirofiban	Control	Tirofiban	Control	Tirofiban	Control	Tirofiban	Control
Zi et al. 2023	606	571	68.0 (58.0–75.0)	68.0 (59.0–76.0)	62.5	65.3	90 days	62.08	67.19	31.29	34.03	8.25	9.45	26.73	29.24	35.14	32.92
Torgano et al. 2010	75	75	71.8 ± 13.7	73.8 ± 8.9	48	49	90 days	65	25	36	41	—	—	11	22	17	27
Siebler et al. 2011	131	129	67.6 (range of 34–81)	65.8 (30–82)	56	62	5 months	34	39	22	29	—	—	18	16	—	—
Qiu et al. 2022	463	485	68 (IQR, 58–74)	67 (IQR, 57–75)	56.8	60.6	90 days	54.2	56.3	16	12	15.3	18.1	21.4	21.7	21.6	25.2
Yu et al. 2022	134	133	68 (38–85)	71 (42–85)	64.2	58.6	90 days	67.9	60.9	99	100	11.2	16.5	34.3	27	—	—
Han et al. 2022	190	190	67 (IQR, 59–75)	67 (IQR, 59–75)	65	70	90 days	68.9	72.2	—	—	25.78	31.05	29.4	27.3	—	—
Du et al. 2022	63	60	65.85 (± 9.62)	65.23 (± 8.71)	66.67	53.33	90 days	63.49	60	28.57	13.33	11.11	11.11	28.57	36.67	42.86	40
Zhang et al. 2022	59	14	69.24 ± 14.88	68.21 ± 12.86	54.2	50	90 days	76.27	78.57	23.73	21.43	27.12	0	16.95	28.57	47.46	21.43
Tao et al. 2025	414	418	68.93 ± 2.86	68.93 ± 2.86	62.8	64.8	90 days	82.6	80.4	12.3	13.9	11.8	9.8	22.0	23.9	19.8	23.4

### 3.3 Quality Assessment of the Included Studies

Out of a total of 9 studies, 4 were assessed to have a low risk of bias, 3 were assessed to have some concerns of bias, and 2 RCTs were judged to have a high risk of bias. Two studies (Yu et al. 2022; Du et al. 2022) had a high risk of bias due to no information about allocation concealment and statistical analysis plans. The studies with some concerns of bias had a lack of information about randomization, allocation concealment, and deviations from the intended intervention. The risk of bias figure is presented in Figure S1.

### 3.4 Outcomes

#### 3.4.1 Functional Independence (mRS = 0–2)

**FIGURE 2:**
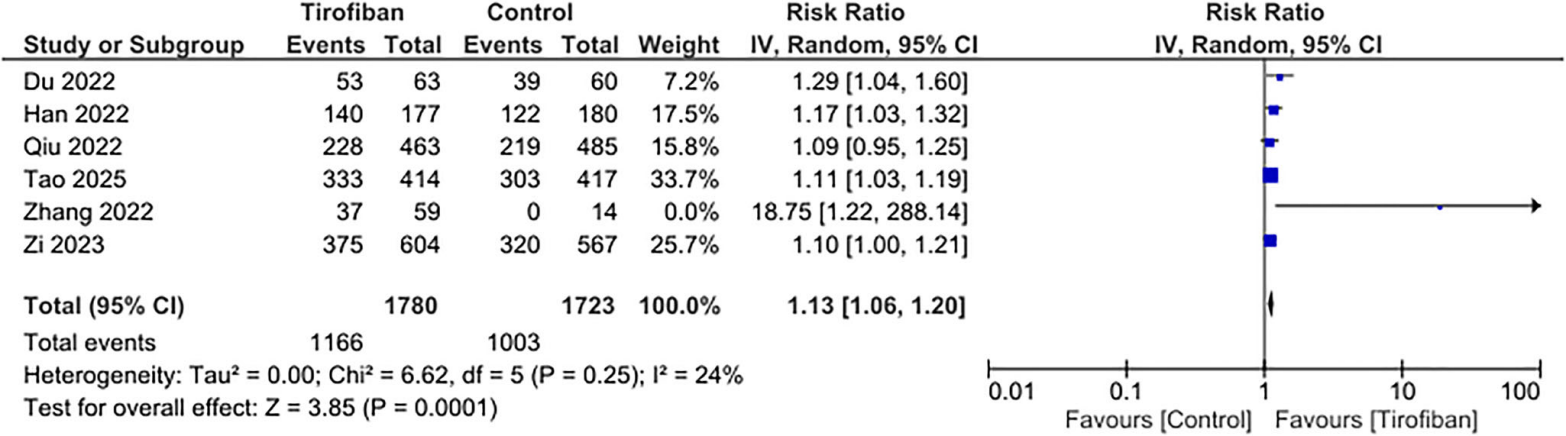
Comparison of functional independence (mRS = 0–2) between patients receiving tirofiban or standard treatment. IV, inverse variance; mRS, Modified Rankin Score.

A total of six studies reported functional independence at 90 days. There was a significant increase in functional independence at 90 days, measured by a modified Rankin Score (mRS) of 0–2 in the tirofiban group compared to the control group (RR 1.13; CI: 1.06–1.20). The heterogeneity (*I* ^2^ = 24%) was low (Figure 2). The overall quality of evidence was rated as moderate due to concerns about risk of bias (Table 3).

**TABLE 2:** Clinical characteristics of included studies.

Study	History of anticoagulation		History of antiplatelet use		NIHSS score, mean ± SD or median (IQR)		Localization of presenting deficit n (%)		
	Tirofiban	Control	Tirofiban	Control	Tirofiban	Control		Tirofiban	Control
Zi et al. 2023	1	0	20	21	9.0 (7.0–10.0)	9.0 (7.0–10.0)	Anterior circulation	489 (80.7)	456 (79.9)
							Posterior circulation	92 (15.2)	94 (16.5)
							Anterior circulation plus posterior circulation	5 (0.8)	7 (1.2)
							Unknown	20 (3.3)	14 (2.5)
Torgano et al. 2010	—	—	28	37	9 (6–16)	9 (7–14)	Total anterior circulation infarct	28 (37)	23 (31)
							Partial anterior circulation infarct	21 (28)	30 (40)
							Lacunar cerebral infarct	21 (28)	19 (25)
							Posterior circulation infarct	5 (7)	3 (4)
Siebler et al. 2011	—	—	85	96	6.0 (4–18)	6.0 (4–18)	—	—	—
Qiu et al. 2022	7.8	7.4	Single antiplatelet therapy: 7.1; dual antiplatelet therapy: 2.2	Single antiplatelet therapy: 8.7; dual antiplatelet therapy: 0	16 (IQR, 12–19)	16 (IQR, 12–20)	Intracranial internal carotid artery	96 (20.7)	98 (20.2)
							Middle cerebral artery segment M1	305 (65.9)	310 (63.9)
							Middle cerebral artery segment M2	62 (13.4)	77 (15.9)
Yu et al. 2022	—	—	—	—	5 (3–19)	6 (3–20)	Anterior	84 (62.7)	74 (55.6)
							Posterior	40 (29.8)	47 (35.3)
							Anterior and posterior	10 (7.5)	12 (9.0)
Han et al. 2022	—	—	—	—	6	5	LAA	72 (40.7)	86 (47.8)
							SVO	105 (59.3)	94 (52.2)
Du et al. 2022	—	—	100	100	2 (1–4)	2 (1–2)	LAA	41 (65.08)	43 (71.67)
							SAO	22 (39.42)	17 (28.33)
Zhang et al. 2022	—	—	23.73	14.27	8.90 ± 2.75 AND 5.93 ± 1.24	8.14 ± 3.51 and 5.29 ± 1.54	LAA	61.02%	85.71%
							SVO	38.98%	14.29%
Tao et al. 2025	—	—	—	—	6 (5–9)	6 (5–9)	Middle cerebral artery segment M1	24 (17.6)	17 (10.8)
							Middle cerebral artery segment M2	12 (8.8)	14 (8.9)
							Internal carotid artery	7 (5.1)	9 (5.7)
							Basilar artery	1 (0.3)	3 (1.9)
							Vertebral artery	5 (3.7)	2 (1.3)
							Anterior cerebral artery	3 (2.2)	5 (3.2)
							Posterior cerebral artery	4 (2.9)	13 (8.3)

LAA: large artery atherosclerosis; SVO: small vessel occlusion.

**TABLE 3:** Grading of recommendation assessment, development, and evaluation (GRADE) summary of findings.

Outcome	No. of participants (studies)	Effect estimate (95% CI)	Risk of bias	Inconsistency	Indirectness	Imprecision	Publication bias	Quality of evidence (GRADE)
**Functional independence (mRS 0–2)**	3503 (Winter and Juergens 2008)	1.13 (1.06–1.20)	Serious	Not serious	Not serious	Not serious	Undetected	⊕⊕⊕⊖ Moderate
**Excellent outcome (mRS 0–1)**	3440 (Winter and Juergens 2008)	1.17 (1.05–1.29)	Serious	Not serious	Not serious	Not serious	Undetected	⊕⊕⊕⊖ Moderate
**Mortallity**	4176 (Siebler et al. 2011)	0.94 (0.63–1.40)	Serious	Not serious	Not serious	Serious	Undetected	⊕⊕⊖⊖ Low
**Any ICH**	3841 (Qiu et al. 2022)	1.25 (1.06–1.47)	Serious	Not serious	Not serious	Not serious	Undetected	⊕⊕⊕⊖ Moderate
**sICH**	3473 (Qiu et al. 2022)	1.48 (0.98–2.24)	Serious	Not serious	Not serious	Serious	Undetected	⊕⊕⊖⊖ Low

#### 3.4.2 Excellent Outcome (mRS = 0–1)

Six studies reported excellent outcome measures. There was a significant increase in excellent outcome at 90 days, measured by a modified Rankin Score (mRS) of 0–1 between the tirofiban group and control group (RR 1.17; CI: 1.05–1.29). The heterogeneity (*I* ^2^ = 40%) was moderate (Figure S2). The overall quality of evidence was rated as moderate due to concerns about risk of bias (Table 3).

#### 3.4.3 Symptomatic ICH (sICH)

**FIGURE 3:**
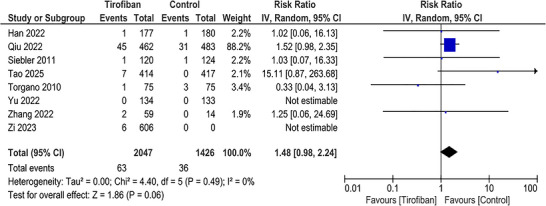
Comparison of symptomatic intracranial hemorrhage (sICH) between patients receiving tirofiban or standard treatment. IV, inverse variance.

There was no significant difference in sICH in the tirofiban group and control group (RR 1.48; CI: 0.98–2.24) (Figure 3). The heterogeneity was minimal (0%). The overall quality of evidence was rated as low due to concerns about inconsistency and risk of bias (Table 3).

### 3.5 Mortality

According to our analysis of data from all 9 RCTs, there was no significant difference regarding mortality between the tirofiban group and control group (RR 0.94; CI: 0.63–1.40). The heterogeneity was moderate (*I* ^2^ = 45%) (Figure S4). The overall quality of evidence was rated as low due to concerns about inconsistency and risk of bias (Table 3).

#### 3.5.1 Any Intracranial Hemorrhage (ICH)

Tirofiban significantly increased the incidence of intracranial hemorrhage as compared to the standard management (RR 1.25; CI: 1.06–1.47) (Figure S3). The heterogeneity was minimal (0%). The overall quality of evidence was rated as moderate due to concerns about risk of bias (Table 3).

## 4 Discussion

This meta‐analysis of 4210 patients from nine RCTs sought to evaluate the efficacy and safety of tirofiban for acute ischemic stroke (AIS). Pooled results demonstrated that intravenous tirofiban increased favorable neurologic functional outcomes (mRS score 0–2 and mRS score 0–1) at 90 days in AIS patients. In terms of safety, we found that tirofiban did not significantly increase the risk of symptomatic intracranial hemorrhage (sICH) or mortality within 90 days compared to control. However, it was associated with a significantly higher rate of radiographic intracranial hemorrhage (ICH) (238/1931 vs. 190/1910).

Mixed findings are found in the data from recent RCTs on intravenous tirofiban for stroke. The two studies with the largest sample sizes (i.e., the RESCUEBT and RESCUEBT2 trials) showed that tirofiban had nonsignificant results in terms of favorable functional outcomes (mRS score 0–2) after 90 days when compared to control (Qiu et al. 2022; Wenjie et al. 2023). Though the tirofiban group was unable to raise positive functional results (mRS score 0–2) in the RESCUEBT2 trial, there was a significant rise in excellent functional outcomes (mRS score 0–1) after 90 days. On the other hand, smaller‐scale studies tend to show that tirofiban greatly enhances favorable functional outcomes (Yu et al. 2022; Han et al. 2022; Zhang et al. 2022; Du et al. 2022).

In terms of safety outcomes, the two largest RCTs revealed that tirofiban was linked to an elevated risk of sICH (RESCUEBT2 trial) or any ICH (RESCUEBT trial). Meanwhile, no significant difference in both sICH and any ICH was seen in the smaller‐scale studies (Torgano et al. 2010; Yu et al. 2022; Han et al. 2022). Tirofiban also had no effect on the 90‐day mortality as reported in the large‐scale studies (Qiu et al. 2022; Wenjie et al. 2023), while more favorable findings were seen in smaller trials like those by Han et al. (2022) and Siebler et al. (2011), wherein tirofiban was found to significantly decrease mortality rates.

These inconsistencies were reconciled by the pooled effect estimates in our meta‐analysis. Heterogeneity was absent in studies concerning any ICH and sICH, which strengthens the evidence for tirofiban causing an increased risk of radiographic ICH without drastically leading to a poor prognosis attributed to sICH. All of the included RCTs in our study administered tirofiban intravenously, and it may have contributed to the insignificant difference in the incidence of sICH, as Gong et al.’s (2020) meta‐analysis specifically noted a rise in fatal ICH only with intra‐arterial tirofiban administration.

Our findings are broadly consistent with those of a previous meta‐analysis (Kamran et al. 2025) that evaluated the efficacy and safety of tirofiban in acute ischemic stroke. Similar to our results, that study reported an improvement in favorable functional outcomes (mRS 0–2) with tirofiban and found no statistically significant difference in symptomatic intracranial hemorrhage or mortality between treatment groups. However, several methodological differences exist between the analyses. The prior meta‐analysis included both randomized and observational studies, whereas our study focused exclusively on randomized controlled trials to minimize confounding and improve the overall quality of evidence. Additionally, our analysis incorporates more recent evidence, including the latest randomized trial by Tao et al. (2025), which was not included in the earlier review. These differences in study design and inclusion criteria may partly explain variations in the magnitude of the pooled effect estimates across analyses.

The safety profile of tirofiban in our study is most consistent with the recent meta‐analysis by Al‐Salihi et al. (2023). This is because both studies exclusively analyzed data from RCTs, whereas prior meta‐analyses incorporated observational, retrospective, and non‐randomized studies. In contrast to earlier reports, our updated analysis similarly demonstrates a significant increase in excellent functional outcomes (mRS 0–1 at 90 days), consistent with the findings of Al‐Salihi et al. Importantly, our results are strengthened by the inclusion of two additional RCTs, most notably the RESCUE‐BT2 trial, the largest and most rigorously designed RCT evaluating tirofiban in acute ischemic stroke to date, which was not available at the time of the prior meta‐analysis, as well as the ASSETT‐IT trial. The incorporation of these trials enhances the robustness and contemporary relevance of our efficacy estimates.

Our study's safety outcomes were in opposition to the findings of meta‐analyses that focused on tirofiban in the context of endovascular therapy (EVT), such as those by Wang et al. (2024) and Zhang et al. (2022), who both reported that tirofiban lowers mortality without influencing sICH or any other ICH. Meanwhile, Liu et al. (2023), Zhou et al. (2020), and Xie et al. (2024) did not show a survival benefit with tirofiban yet still supported its safety, with results not revealing any significant changes in mortality, sICH, and any ICH.

In terms of efficacy estimates, each of the previous meta‐analyses was consistent with our study except Fu et al. and Gong et al., in showing that tirofiban increased the proportion of patients who achieved mRS 0–2 scores. Only Zhou et al. demonstrated that tirofiban enhances mRS 0–1 scores. Liu et al. and Zhou et al. categorized their studies into two distinct groups. Liu evaluated the effects of tirofiban administration in patients with and without mechanical EVT after intravenous thrombolysis (IVT) and found that functional outcomes considerably improved when tirofiban was administered following IVT alone, but not in the group that also received EVT. Zhou et al. divided their studies into one group receiving tirofiban monotherapy and the other receiving tirofiban combined with IVT. Meanwhile, our meta‐analysis only included one study that assessed the effect of tirofiban before EVT (Qiu et al. 2022), while one other study evaluated the effect of tirofiban following IVT (Zhang et al. 2022).

Tirofiban, a rapidly acting and highly selective inhibitor of the glycoprotein (GP) IIb/IIIa receptor, distinguishes itself from other drugs used for AIS by its reversible binding and short half‐life of 2 h (Wenjie et al. 2023; Zhou et al. 2020 Feb). This characteristic allows bleeding time to normalize within approximately 3 h after discontinuation, contrasting with the higher bleeding complications associated with drugs like aspirin due to irreversible binding (Zinkstok and Roos 2012), and those like abciximab due to their longer half‐lives (Adams et al. 2008). Nevertheless, tirofiban does not increase survival in AIS patients, according to our pooled analysis, even though it may improve neurologic function. The increased incidence of any ICH underscores the need for careful assessment of hemorrhagic risk while using tirofiban in clinical practice. More multicentric RCTs are still necessary to determine the best balance between bleeding risk and functional improvement and to more thoroughly assess tirofiban's safety and efficacy in more targeted treatment contexts and stroke subtypes.

Our meta‐analysis differs from most of its predecessors in that it only includes data from RCTs, providing the strongest evidence for our conclusions. Notably, the intravenous route of tirofiban administration in all of our included studies excludes any possibility that different administration methods and pharmacokinetics could have affected the results. Additionally, our analysis combined data from trials with different stroke vessel sizes and subtypes, such as those that included large‐vessel occlusions only (RESCUEBT trial) or those that excluded them completely (SaTIS trial and RESCUEBT2 trial). This makes it possible to apply the results of our study to a wide range of stroke variants and presentations. Furthermore, the possibility of overestimating treatment effects—which is frequently observed in meta‐analyses restricted to smaller studies—is reduced by our inclusion of both small‐ and large‐scale trials.

Except for the SETIS and SaTIS trials, all studies included in this meta‐analysis were conducted in China. Due to this, the study's generalizability to the global population is limited, as it is uncertain whether the therapeutic efficacy of tirofiban similarly applies to non‐Chinese stroke patients. Uniformity was also further decreased due to the differing dosages of tirofiban administered across these trials as well as the varying placebos used. While some trials, like the RESCUE BT and SaTIS trials, used saline as the control, others, like the ESCAPIST trials, chose to use aspirin. The SETIS trial opted to use no placebo at all. Several studies exhibited “some concerns” or an outright “high risk of bias,” with the latter mainly due to performance and detection biases. Low to moderate heterogeneity was also present in some of the outcomes. The definition of sICH varied across the included studies, with different trials using established criteria such as NINDS, ECASS II, and SITS‐MOST criteria. These definitions differ in their diagnostic thresholds and clinical criteria, which may influence the reported incidence of sICH. Therefore, the pooled estimate for this outcome should be interpreted with caution. As such, any conclusions and recommendations informed by this data must be made with an awareness of these limitations. More large‐scale RCTs are needed to better ascertain the role of tirofiban in acute ischemic stroke.

## 5 Conclusion

This meta‐analysis suggests that tirofiban may improve favorable functional outcomes (mRS 0–2) in acute ischemic stroke, with moderate certainty of evidence. No significant increase in sICH was observed (low certainty), although some analyses showed higher overall intracranial hemorrhage rates. These results should be interpreted cautiously, and further high‐quality randomized trials are needed to clarify its efficacy and safety before routine guideline adoption.

## Author Contributions

**Dorothy Lim Bullecer**: Investigation, Writing – Original Draft, Writing – Review & Editing, Visualization, Methodology, Formal Analysis, Resources, **Cara Mohammed**: Investigation, Writing – Original Draft, Writing – Review & Editing, Validation, Formal Analysis, Resources, **Julie Anne De Lima Loiola**: Writing – Original Draft, Writing – Review & Editing, Investigation, Methodology, Formal Analysis, Data Curation, **Mary Gabrielle Te Chy**: Data Curation, Formal Analysis, Writing – Review & Editing, Writing – Original Draft, Investigation, Validation, **Samratul Fuadah Ramli**: Visualization, Writing – Original Draft, Investigation, Writing – Review & Editing, Formal Analysis, Data Curation, **Abimbola Sodiq Hussein**: Data Curation, Software, Methodology, Writing – Review & Editing, Visualization, Investigation, Writing – Original Draft, **Wasid Bin Showkat**: Investigation, Writing – Original Draft, Writing – Review & Editing, Validation, Methodology, Formal Analysis, Data Curation, **Thahsin Taikadan**: Resources, Formal Analysis, Visualization, Writing – Review & Editing, Writing – Original Draft, Investigation, **Sarika Mutyala**: Writing – Original Draft, Writing – Review & Editing, Visualization, Software, Resources, **Muhammad Ayyan**: Supervision, Resources, Project Administration, Formal Analysis, Visualization, Writing – Review & Editing, Validation, Methodology, Conceptualization, Writing – Original Draft, Investigation, **Muhammad Aemaz Ur Rehman**: Investigation, Writing – Original Draft, Writing – Review & Editing, Methodology, Project Administration, Supervision, **Adeel Ahmad**: Supervision, Project Administration, Writing – Review & Editing, Writing – Original Draft, **Asma'a Munasar Ali Alsubari**: Writing – Original Draft, Funding Acquisition, Investigation, Writing – Review & Editing, Project Administration, Supervision

## Funding

The authors have nothing to report.

## Ethics Statement

No ethical approval was required for this study.

## Consent

No consent was required for this study.

## Conflicts of Interest

The authors declare that they have no conflicts of interest and no financial interests related to the material of this manuscript.

## Supporting information




**Supplementary Information**: brb371520‐sup‐0001‐SuppMat.docx

## Data Availability

The data that supports the findings of this study are available in the supplementary material of this article
