# Supplementary material for: Efficacy and Safety of Tirofiban for the Management of Acute Ischemic Stroke: A Systematic Review and Meta‐Analysis of Randomized Controlled Trials (RCTs)
Source: Brain Behav. 2026 May 31;16(6):e71520. doi: 10.1002/brb3.71520 (PMC13240329; doi:10.1002/brb3.71520)
Supplement: Supplementary file 1 — Supplementary Information: brb371520‐sup‐0001‐SuppMat.docx [file BRB3-16-e71520-s001.docx]

**Supplementary File**

**Efficacy and Safety of Tirofiban for the Management of Acute Ischemic Stroke: A Systematic Review and Meta-Analysis of Randomized Controlled Trials (RCTs)**

**Figure S1. Risk of bias analysis of included studies using Cochrane’s Risk of bias tool (RoB 2.0)**


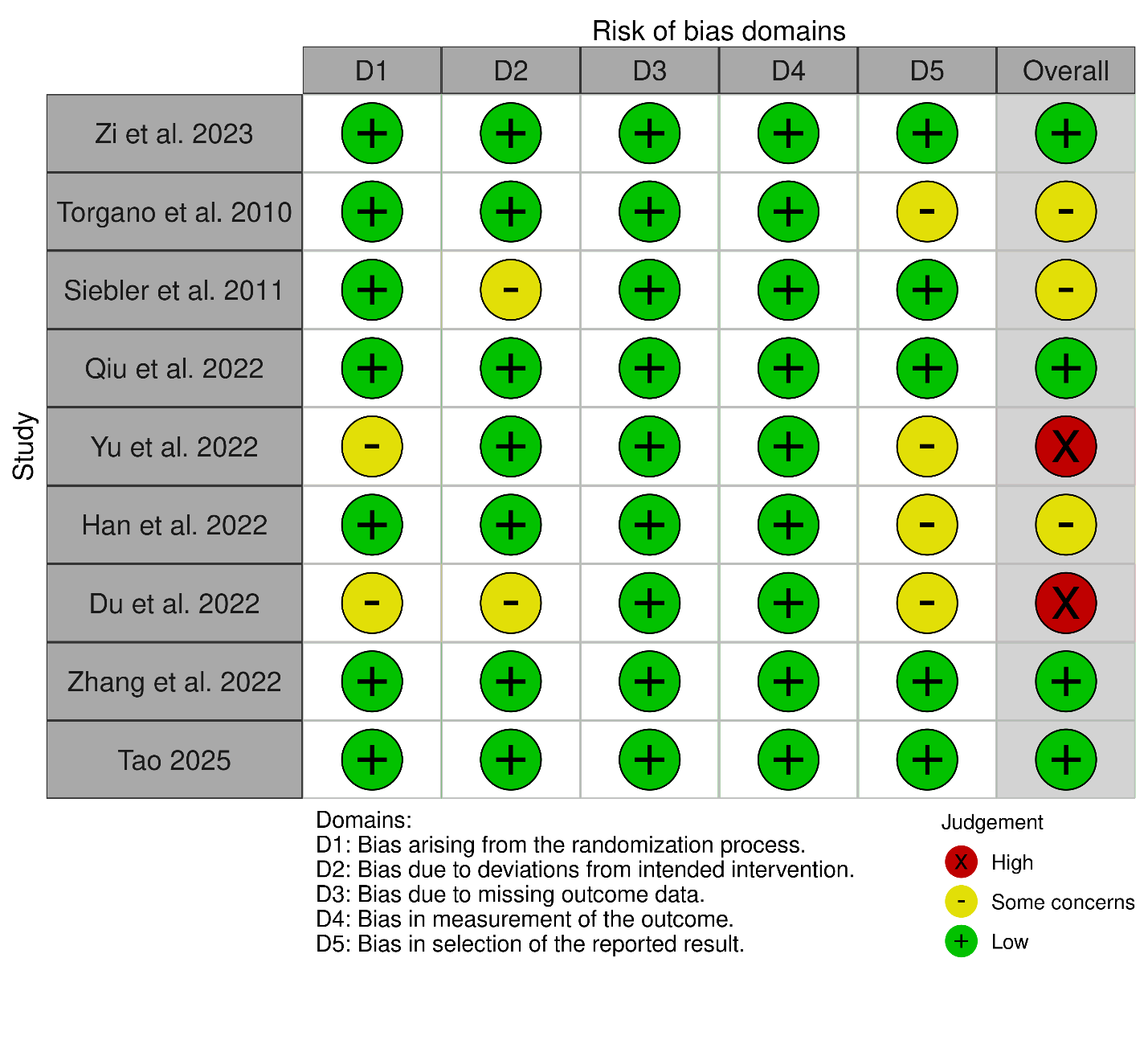


**Figure S2. Comparison of Excellent Outcome (mRS = 0-1) between patients receiving Tirofiban or standard treatment. IV, inverse variance; mRS, Modified Rankin Score.**


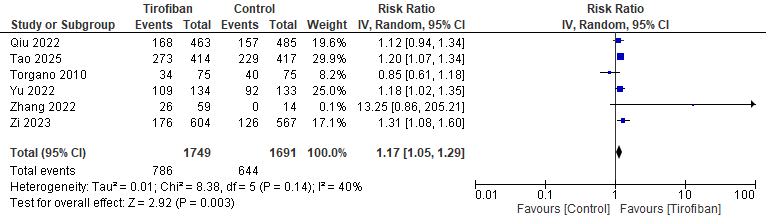


**Figure S3. Comparison of any Intracranial Hemorrhage (ICH) between patients receiving Tirofiban or standard treatment. IV, inverse variance.**


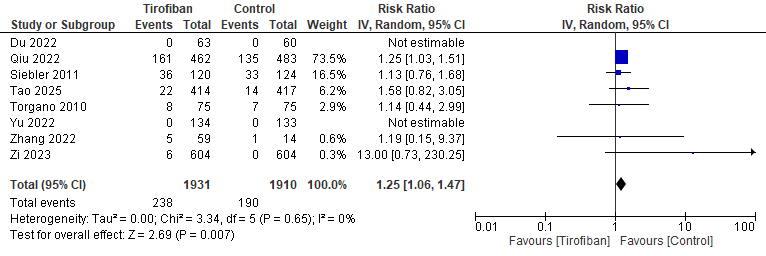


**Figure S4. Comparison of mortality between patients receiving Tirofiban or standard treatment. IV, inverse variance.**


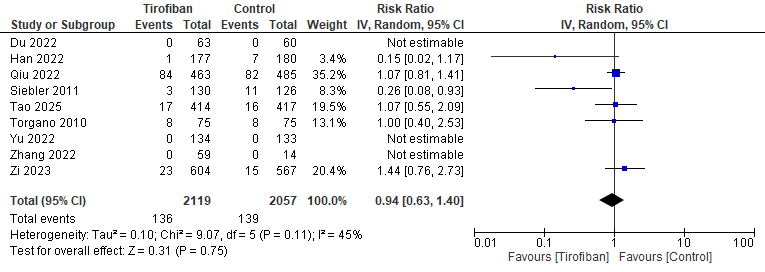


**Supplementary Table 1. Search Strategy for MEDLINE (PubMed Format)**

| **Number** | **Search Items** |
| --- | --- |
| #1 | "ischemic stroke"[MeSH Terms] OR ("ischemic"[All Fields] AND "stroke"[All Fields]) OR "ischemic stroke"[All Fields] OR ("acute"[All Fields] AND "ischemic"[All Fields] AND "stroke"[All Fields]) OR "acute ischemic stroke"[All Fields] |
| #2 | (tirofiban OR aggrastat OR Agrastat) |
| #3 | randomized OR randomised OR random OR randomly OR 'clinical trial' |
| #4 | #1 AND #2 AND #3 |
